# Omicron BA.1 and BA.2 immune response in naïve and prior infected persons

**DOI:** 10.1101/2022.04.07.22273565

**Published:** 2022-04-10

**Authors:** Ludwig Knabl, Hye Kyung Lee, Mary Walter, Priscilla A. Furth, Lothar Hennighausen

**Affiliations:** 1 TyrolPath Obrist Brunhuber GmbH, Zams, Austria.; 2 National Institute of Diabetes, Digestive and Kidney Diseases, National Institutes of Health, Bethesda, MD 20892, USA.; 3 Clinical Core, National Institute of Diabetes, Digestive and Kidney Diseases, National Institutes of Health, Bethesda, MD 20892, USA.; 4 Departments of Oncology & Medicine, Georgetown University, Washington, DC, USA.

## Abstract

The highly transmissible SARS-CoV-2 Omicron (B.1.1.529) variant has replaced previous variants and is less susceptible to neutralizing antibodies elicited by vaccination or infection^1–3^. Currently, BA.2 is the dominant omicron sublineage^4^. Vaccinated individuals with BA.1 infection develop measurable neutralizing antibody titers against BA.1 and BA.2^5^. The ability of BA.2 infection to induce neutralizing antibodies in unvaccinated individuals, either without or with previous SARS-CoV-2 infection, is pending definition.

We measured antibody titers and neutralizing antibody responses in 74 outpatients infected with either omicron BA.1 or BA.2 sublineages. While none of the patients had been vaccinated, 26 had been infected by an earlier variant. Demographic and clinical information are provided in Table S1 (Supplementary Appendix). More than 20% of the BA.1 infected, but none of the BA.2 infected patients, had moderate or severe disease.

Humoral response was measured between 7 and 21 days post documented omicron BA.1 or BA.2 infection. In previously uninfected and unvaccinated individuals both anti-omicron and ancestral spike IgG titers were 4–5-fold lower following BA.2 as compared to BA.1 infection (Fig. 1A). In contrast, antibody titers in individuals previously infected with an earlier SARS-COV-2 variant were 11–86-fold higher, with no difference in titers between those infected with the BA.1 versus BA.2 variants. For reference, titers from individuals infected with the Beta variant are shown. These approximate those seen in individuals previously infected with an earlier variant who then experienced BA.1 or BA.2 infection. The differential response in antibody generation following BA.1 and BA.2 infection was consistent across all SARS-COV-2 variants tested (Fig. 1B).

We next measured neutralization capacity using the angiotensin-converting enzyme 2 (ACE2) binding inhibition assay (Fig. 1C). Neutralizing activities against the ancestral strain and omicron were similarly low in antigen naïve individuals infected with either BA.1 or BA.2. Similar to overall antibody titers, neutralizing activity was higher in those previously infected. Activity from BA.1 and BA.2 infected individuals approximated that found for individuals infected with the Beta variant and was similar across all SARS-COV-2 variants tested against (Fig. 1D).

These data reveal that in antigen naïve individuals the immunologic response following Omicron BA.2 infection is even lower than BA.1 infection. One could postulate that the lower neutralization response following BA.2 infection might contribute to prolonged circulation in the population due to attenuated protection from re-infection. Potentially, this would be abrogated by vaccination following resolution of the omicron infection per current guidelines.

## Methods

### Ethics

This study was approved (EK Nr: 1064/2021) by the Institutional Review Board (IRB) of the Office of Research Oversight/Regulatory Affairs, Medical University of Innsbruck, Austria, which is responsible for all human research studies conducted in the State of Tyrol (Austria). Participant information was coded and anonymized.

### Study population, study design and recruitment

A total of 74 patients infected with Omicron were recruited for the study under informed consent. All with no vaccination and one-third with history of prior infection (Table S1). Recruitment and blood sample collection took place between December 2021 and March 2022. This study was approved (EK Nr: 1064/2021) by the Institutional Review Board (IRB) of the Office of Research Oversight/Regulatory Affairs, Medical University of Innsbruck, Austria, which is responsible for all human research studies conducted in the State of Tyrol (Austria). The investigators do not need to have an affiliation with the University of Innsbruck. A waiver of informed consent was obtained from the Institutional Review Board (IRB) of the Office of Research Oversight/Regulatory Affairs, Medical University of Innsbruck (https://www.i-med.ac.at/ethikkommission/). **Written informed consent was obtained from all subjects.** This study was determined to impose minimal risk on participants. All methods were carried out in accordance with relevant guidelines and regulations. All research has been have been performed in accordance with the Declaration of Helsinki (https://www.wma.net/policies-post/wma-declaration-of-helsinkiethical-principles-for-medical-research-involving-human-subjects/). In addition, we followed the ‘Sex and Gender Equity in Research – SAGER – guidelines’ and included sex and gender considerations where relevant.

### Antibody assay

End-point binding IgG antibody titers to various SARS-CoV-2–derived antigens were measured using the Meso Scale Discovery (MSD) platform. SARS-CoV-2 spike, nucleocapsid, Alpha, Beta, Gamma, Delta, and Omicron spike subdomains were assayed using the V-plex multispot COVID-19 serology kits (Panel 23 (IgG) Kit, K15567U). Plates were coated with the specific antigen on spots in the 96 well plate and the bound antibodies in the samples (1:50000 dilution) were then detected by anti-human IgG antibodies conjugated with the MSD SULPHO-TAG which is then read on the MSD instrument which measures the light emitted from the tag.

### ACE2 binding inhibition (Neutralization) ELISA

The V-PLEX COVID-19 ACE2 Neutralization kit (Meso Scale Discovery, Panel 23 (ACE2) Kit, K15570U) was used to quantitatively measure antibodies that block the binding of ACE2 to its cognate ligands (SARS-CoV-2 and variant spike subdomains). Plates were coated with the specific antigen on spots in the 96 well plate and the bound antibodies in the samples (1:10 dilution) were then detected by Human ACE2 protein conjugated with the MSD SULPHO-TAG which is then read on the MSD instrument which measures the light emitted from the tag.

### Statistical analysis

For comparison of samples, data were presented as standard deviation in each group and were evaluated with 2-way ANOVA followed by Tukey’s multiple comparisons test on GraphPad Prism software (version 9.0.0).

## Supplementary Material

1

## Figures and Tables

**Figure 1.**
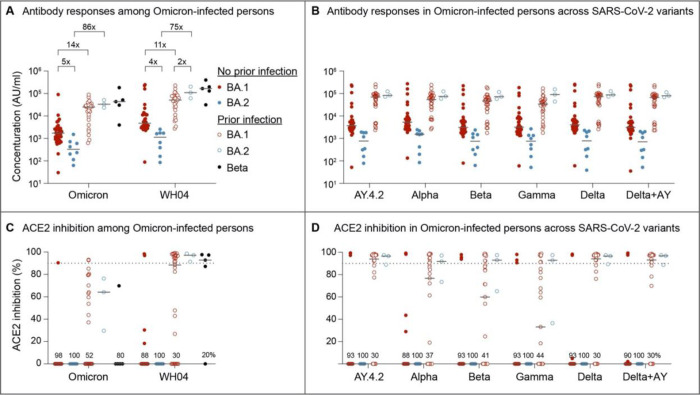
Serology and Neutralizing antibody responses in outpatients infected with the omicron BA.1 and BA.2 sublineages. Panel A and B show plasma IgG antibody binding the SARS-CoV-2 RBD (spike) from the ancestral and Omicron strains (A) as well as other variants (B) in the unvaccinated BA.1 and BA.2 Omicron patients without or with prior SARS-CoV-2 infection. Panel C and D show neutralizing antibody response by measuring inhibition of binding between ACE2 and SARS-CoV-2 spike protein. Antibody titers were measured 1–3 weeks after the infection. Results are shown as the median and dots for each data (No prior infection, BA.1, *n* = 40; No prior infection, BA.2, *n* = 10; Prior infection, BA.1, *n* = 23; Prior infection, BA.2, *n* = 3; Beta infection, *n* = 4).
