# Supplementary material for: Omicron BA.1 and BA.2 immune response in naïve and prior infected persons
Source: medRxiv. 2022 Apr 10:2022.04.07.22273565. Preprint. [Version 1] doi: 10.1101/2022.04.07.22273565 (PMC9016656; doi:10.1101/2022.04.07.22273565)
Supplement: Supplementary file 1 [file NIHPP2022.04.07.22273565V1-supplement-1.pdf]

medRxiv preprint doi: https://doi.org/10.1101/2022.04.07.22273565; this version posted April 10, 2022. The copyright holder for this preprint (which was not certified by peer review) is the author/funder, who has granted medRxiv a license to display the preprint in perpetuity. This article is a US Government work. It is not subject to copyright under 17 USC 105 and is also made available for use under a CC0 license.

Table S1. Characteristics of Omicron study population.

	No prior infection		Prior infection		Chi-Square
	BA.1	BA.2	BA.1	BA.2	
N	40	10	21	3	
**Age** (years), median (range)	42 (9-83)	40 (13-65)	33 (10-63)	33 (28-43)	23.68
**Gender**					
Female	31 (78%)	5 (50%)	11 (52%)	2 (67%)	1.10
Male	9 (22%)	5 (50%)	10 (48%)	1 (33%)	2.56
**Medical condition**	21 (52.5%)	3 (30%)	5 (24%)	1 (33%)	43.82
Auto-immune Disease	1	0	2	0	
Chronic Heart Disease	1	0	0	0	
Chronic Pulmonary Disease	2	0	0	0	
Dementia	1	0	0	0	
Diabetes	1	0	0	0	
Gout	1	0	0	0	
Hypertension	4	0	0	0	
Hypothyroid	7	3	3	1	
Kidney Disease	1	0	0	0	
Multiple Sclerosis	1	0	0	0	
S/P Cancer	1	0	0	0	
**COVID-19 disease severity**					
Asymptomatic	3 (7.5%)	0	0	0	
Mild	15 (37.5%)	6 (60%)	13 (62%)	3 (100%)	2.15
Mild with SOB*	13 (32.5%)	4 (40%)	6 (29%)	0	
Moderate	7 (17.5%)	0	2 (10%)	0	
Severe	2 (5%)	0	0	0	
**Days from positive PCR test to sampling, median (range)**					
Sampling	14 (7-21)	14 (7-17)	13 (7-18)	14 (7-19)	19.82

*SOB, shortness of breath.
